# Editorial: Eye movement tracking in ocular, neurological, and mental diseases

**DOI:** 10.3389/fnins.2024.1364078

**Published:** 2024-01-19

**Authors:** Yuexin Wang, Xuemin Li

**Affiliations:** ^1^Department of Ophthalmology, Peking University Third Hospital, Beijing, China; ^2^Beijing Key Laboratory of Restoration of Damaged Ocular Nerve, Beijing, China

**Keywords:** eye movement, eye tracking, ocular disease, neurological disease, mental disease, dynamic vision

Eye movement and dynamic vision abnormalities could be seen in various ocular, neurological, and mental disorders. Therefore, eye movement tracking and accurate evaluation and interpretation of eye movement patterns are significant to disease assessment, diagnosis and treatment. Now, eye-tracking-based tasks and dynamic vision can effectively be integrated into visual and cognitive function evaluation that contributes to assessing several ocular and mental diseases. To further promote the standardized application of the eye movement tracking-based assessment paradigm, the relationship between eye movement patterns and disease parameters should be elucidated qualitatively and quantitatively. The present Research Topic contains a representative collection of studies regarding eye movement tracking and dynamic vision and its interpretation in various ocular and mental disorders.

The majority of the articles in the Research Topic combine various tasks and measurements of eye movement in patients with neurological or mental diseases. The coordination between eye movement and daily tasks might be affected in patients with these disorders. Terao et al. investigated the role of the cerebellum and basal ganglia in eye-voice coordination during reading aloud in patients with Parkinson's disease and spinocerebellar degeneration (SCD). They found that SCD patients have restricted ability to advance text processing ahead of the gaze due to slowed vocal output, and PD patients have slowed scanning but effectively utilize advanced processing of upcoming text. Opwonya et al. developed machine learning-based models for classifying mild cognitive impairment (MCI) using eye movement data during prosaccade/antisaccade and go/no-go tasks. The models achieved an outstanding performance combining eye movement metrics, demographics, and cognitive test scores for MCI classification, and they found that changes in eye movement metrics in MCI are mainly attentional and executive function deficits. Ni et al. explored the influence of autistic traits and social anxiety on different temporal stages of attention to the eyes in college students, and their movement was recorded during the virtual face viewing. The study suggested the separate and interactive roles of autistic traits and social anxiety in ocular attention that highlight the application of eye-tracking techniques in psychiatric diagnosis. Calancie et al. enrolled pediatric patients with borderline personality disorder (BPD) symptoms with and without a comorbid attention deficit and hyperactivity disorder (ADHD) diagnosis and quantified their temporal learning and performance to predictable or unpredictable stimuli in metronome tasks combined with video-based eye tracking. The research supports normal temporal motor prediction in patients with BPD and a reduced response inhibition in BPD combined with ADHD. Meng et al. applied eye movement patterns to cartoon characters or real persons in identifying children with autism spectrum disorder (ASD) and found that machine learning algorithms provide promising results in identifying ASD with eye movement metrics. Woo et al. investigate the relationship between attentional bias for food cues and hunger in individuals with binge eating problems with the eye tracker. The research demonstrated that attentional bias for high-calorie food occurred in individuals with binge eating problems without awareness.

As for ocular diseases, conventional visual function assessment mainly focuses on static vision. Dynamic vision, involving the coordination of complicated eye movement, is limited evaluated in clinical ophthalmology. Wang, Guo et al. investigated the impact of different corneal refractive surgeries on dynamic visual acuity (DVA) and found that postoperative DVA was better than before the surgery in adult myopic patients. Ren et al. examined DVA in dry eye patients and demonstrated that DVA is significantly associated with symptoms and signs of dry eye disease. The patients with corneal fluorescein staining and severe meibomian gland dropout have worse DVA than those without the signs. These studies demonstrate that DVA is a promising indicator for functional vision in patients with ocular diseases or surgeries that might better reflect the vision in real-life scenarios.

Conventionally, eye-tracking research presents visual tasks on a two-dimensional screen. With the development of virtual reality (VR), it could present three-dimensional objects in versatile surroundings and simultaneously record eye movement. In the Research Topic, two studies applied VR technology in eye movement research. Fan et al. explored the eye movement characteristics during virtual reality games of primary mode or 360 modes. They found that the saccade duration and amplitude were greater in the mode with visual stimulus from all around the subjects, and the primary mode is more likely to cause visual fatigue. Tang et al. designed a virtual reality-based mental rotation task using 3D stimuli and applied an eye tracker to record eye movement during task. The result showed that the fixation time and number of within-object fixation and saccades for mirrored objects were lower than that for identical objects.

Outstanding dynamic vision and accurate object tracking are crucial in some occupations, including driver, pilot and athletes. As shown in the Research Topic, Liu et al. enrolled pilots to complete visual search tasks with various clutter factors using an eye tracker to record the viewing path in a timely manner. The research found a significant difference among eye movement metrics at different clutter and developed a quantitative measurement for declutter design and appraisal of cockpit displays. Besides the original articles, the Research Topic also includes a review of dynamic vision. Wang, Yu et al. reviewed the dynamic visual and eye movement changes in high-altitude environments. They illustrated that dynamic vision performance gradually decreased as the altitude increased, and the pupil saccade function did not differ under different hypoxic conditions.

The present Research Topic demonstrates that eye movement and dynamic vision evaluation potentially provide important information in the assessment and diagnosis of ocular diseases, neurological diseases, and mental disorders. Further research is required to standardize the paradigm and integrate eye-tracking and dynamic vision evaluation into conventional medical systems to better interpret and understand the disease.

## Author contributions

YW: Conceptualization, Validation, Writing—original draft. XL: Conceptualization, Validation, Writing—review & editing.

